# A Functional *IL22* Polymorphism (rs2227473) Is Associated with Predisposition to Childhood Cerebral Malaria

**DOI:** 10.1038/srep41636

**Published:** 2017-01-31

**Authors:** Sandrine Marquet, Ianina Conte, Belco Poudiougou, Laurent Argiro, Hélia Dessein, Charlène Couturier, Florence Burté, Aboubacar A. Oumar, Biobele J. Brown, Abdoualye Traore, Nathaniel K. Afolabi, Abdoulaye Barry, Samuel Omokhodion, Wuraola A. Shokunbi, Olugbemiro Sodeinde, Ogobara Doumbo, Delmiro Fernandez-Reyes, Alain J. Dessein

**Affiliations:** 1 Aix-Marseille University, INSERM, GIMP, Labex ParaFrap, Marseille, France; 2 Department of Computer Science, Faculty of Engineering Sciences, University College London, Gower Street, London, WCE2 6BT, United Kingdom; 3 Malaria Research and Training Center, Department of Epidemiology of Parasitic Disease, Faculty of Medicine, USTTB, BP 1805, Bamako, Mali; 4 Centre des Oeuvres Universitaires, University of Bamako, BP 1805, Bamako, Mali; 5 Department of Pediatrics, College of Medicine, University of Ibadan, University College Hospital, Ibadan, Nigeria; 6 Department of Hematology, College of Medicine, University of Ibadan, University College Hospital, Ibadan, Nigeria; 7 Childhood Malaria Research Group, College of Medicine, University of Ibadan, University College Hospital, Ibadan, Nigeria; 8 Pediatric Wards, Gabriel Toure Hospital, Bamako, Mali.

## Abstract

Cerebral malaria (CM) is a severe complication of *Plasmodium falciparum* infection. This encephalopathy is characterized by coma and is thought to result from mechanical microvessel obstruction and an excessive activation of immune cells leading to pathological inflammation and blood-brain barrier alterations. IL-22 contributes to both chronic inflammatory and infectious diseases, and may have protective or pathogenic effects, depending on the tissue and disease state. We evaluated whether polymorphisms (*n* = 46) of *IL22* and *IL22RA2* were associated with CM in children from Nigeria and Mali. Two SNPs of *IL22*, rs1012356 (*P* = 0.016, OR = 2.12) and rs2227476 (*P* = 0.007, OR = 2.08) were independently associated with CM in a sample of 115 Nigerian children with CM and 160 controls. The association with rs2227476 (*P* = 0.01) was replicated in 240 nuclear families with one affected child from Mali. SNP rs2227473, in linkage disequilibrium with rs2227476, was also associated with CM in the combined cohort for these two populations, (*P* = 0.004, OR = 1.55). SNP rs2227473 is located within a putative binding site for the aryl hydrocarbon receptor, a master regulator of IL-22 production. Individuals carrying the aggravating T allele of rs2227473 produced significantly more IL-22 than those without this allele. Overall, these findings suggest that IL-22 is involved in the pathogenesis of CM.

Malaria is endemic to more than 90 countries and continues to be a major global health challenge. In 2015, there were an estimated 214 million clinical cases of malaria and 438000 deaths. Most deaths occur among children under the age of five years living in Sub-Saharan Africa. Only 5% of people infected with *P. falciparum* develop severe malaria, and disease outcome is influenced by host genetic factors. Infection with *P. falciparum* may result in severe clinical diseases, such as cerebral malaria, severe malarial anemia, respiratory distress and hemoglobinuria. Cerebral malaria is one of the most severe forms of the disease and is characterized by encephalopathy with loss of consciousness. The sequestration of parasitized red blood cells in the small capillaries of the brain leads to mechanical obstruction, hypoxia, the activation of local immune cells and local inflammation. Independent studies have also suggested functional impairment of the blood-brain barrier (BBB) in the pathophysiology of CM^1^,^2^,^3^.

IL-22, a member of the IL-10 cytokine family, induces the early recruitment of immune cells, stimulates the local production of antimicrobial molecules, and promotes the repair of damaged epithelia and the “anti-LPS” response^4^,^5^. IL-22 acts on non-hematopoietic cells such as epithelia, hepatocytes, and pancreatic cells, and is produced by many different types of lymphocytes of the innate and adaptive immune systems^5^,^6^, including CD4 T cells, such as Th17 and Th22 cells, γδ T cells, natural killer (NK) cells, lymphoid tissue inducer (LTi) cells, and LTi- like cells^5^,^6^,^7^. The heterodimeric receptor for IL-22, composed of IL-22R1 and IL-10R2 chains, is expressed exclusively on non-hematopoietic cells. IL-10R2 is ubiquitously expressed^8^, whereas IL-22R1 expression is limited to tissues such as the skin, liver, lung, kidney, pancreas, and the surface of human BBB endothelial cells^9^. There is also a secreted IL-22-binding receptor, the IL-22 binding protein (IL-22BP), encoded by an independent gene (*IL22RA2*). IL-22BP lacks the intracellular and transmembrane domains of the IL-22R^10^,^11^ but has a high affinity for IL-22. IL-22BP therefore competes strongly with IL-22R for IL-22 binding.

The role of IL-22 in inflammatory and infectious diseases depends on the tissue and the disease. IL-22 protects against acute hepatitis^5^,^12^ and stimulates tissue regeneration in experimental models of liver disease^13^. It protects against liver fibrosis and cirrhosis in humans with chronic liver infections^14^ and has also been associated with protection against human Kala Azar^15^. By contrast, IL-22 plays a pathogenic role in multiple sclerosis by promoting leukocyte infiltration in the brain^9^ and contributes to the pathogenesis of West Nile encephalitis by facilitating the infiltration of the CNS with neutrophils following infection^16^. IL-22 has also been implicated in Guillain-Barré Syndrome, an acute autoimmune-mediated inflammatory demyelinating disease^17^.

We hypothesized that IL-22 might play a role in the response to *Plasmodium falciparum* infection, given the presence of the IL-22R complex at the BBB and the implication of IL-22 in the early immune response to pathogens. We report here genetic evidence for the involvement of IL-22 in cerebral malaria in children.

## Results

### The *IL22* variants rs1012356 and rs2227476 are associated with childhood cerebral malaria

**Figure 1:**
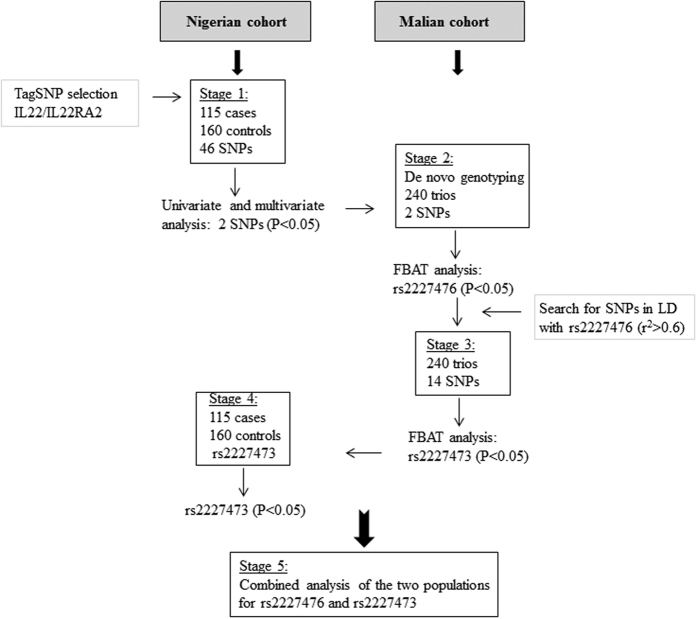
Scheme of the study design. The association study was performed in a Nigerian case-control cohort, with subsequent replication in a family-based Malian population. Two SNPs located in the promoter region of *IL22* were associated with CM in both populations.

We selected 46 TagSNPs within the *IL22* and *IL22RA2* genes and a 5 kb region upstream and downstream from these two genes (Table 1). These SNPs are representative of the correlation bins (r^2^ = 0.8) established with the 1000 Genomes YRI database. We successfully genotyped 40 polymorphisms in the Nigerian population, which consisted of 115 children with cerebral malaria and 160 community controls (stage 1 in Fig. 1). Univariate analysis showed no significant association between CM and the *IL22RA2* polymorphisms tested. The analysis revealed a significant association with disease for *IL22* SNP rs1012356 (bin II) and *IL22* SNP rs2227476 (bin III) (*P* < 0.05) (Table 2). The most significant association was observed for SNP rs2227476 (*P* = 0.018) [OR = 1.85 (1.11–3.08)]. The T allele of rs2227476 was more frequent among cases (32.8%) than controls (23.9%), (*P* = 0.028). We identified a trend towards association with CM for two other polymorphisms from two other bins: rs1179251 (bin I) (*P* = 0.057; OR = 1.61) and rs4913285 (bin IV) (*P* = 0.056; OR = 1.74) (Table 2). Multivariate analysis on SNPs rs1179251, rs1012356, rs2227476, and rs4913285 showed that rs1012356 (*P* = 0.016; OR = 2.12), and rs2227476 (*P* = 0.007; OR = 2.08) were independently associated with CM (Table 2). The other SNPs were not significantly associated with CM. SNPs rs1012356 and rs2227476 are located within the intron and the 5′ region of the *IL22* gene, respectively.

We then evaluated these two SNPs associated with CM in the Nigerian cohort (stage 2 in Fig. 1) in a second cohort consisting of 240 nuclear families from Mali. The association of SNP rs2227476 (2-tailed test; *P* = 0.008) with CM was replicated. The T allele was identified as the risk allele, as in the Nigerian sample (Table 3). Using a case-pseudo control data set and conditional logistic regression, we obtained an estimated OR of 1.56 (1.09–2.17). This association remained significant after permutation testing (*P* = 0.01). We identified a trend towards association with CM for the rs1012356 polymorphism (*P* = 0.06). The rs1012356 SNP was not retained in the model in a multivariate analysis including both SNPs.

### SNP rs2227473, which is in linkage disequilibrium with rs2227476, is also associated with CM in both populations

We then investigated the possible association of additional polymorphisms in linkage disequilibrium with rs2227476 with CM in the Malian sample. We selected all SNPs correlated with rs2227476 (r^2^ > 0.6) in an analysis of the 1000 Genomes YRI database and located within 1 Mb on either side of the rs2227476 polymorphism. We identified 14 SNPs clustered together in 7.3 kb of the 5′ region of *IL22*.

We genotyped the Malian nuclear families for these SNPs (stage 3 in Fig. 1). We excluded one SNP from the Sequenom Plex for genotyping because it did not pass the quality control filter. We found that rs2227473 was also associated with CM in the Malian cohort (2-tailed test; *P* = 0.007). The minor T allele, with a frequency of 0.25, was overrepresented in the children with CM (112 rather than the 94.2 expected in the absence of association), OR = 1.64 (1.15–2.35). We checked that rs2227473 was also in linkage disequilibrium (LD) with rs2227476 (r^2^ = 0.5) in the Malian cohort.

We validated the association of rs2227473 with CM in the case-control cohort from Nigeria, (stage 4 in Fig. 1). This variant was associated with disease in Nigeria (one-tailed test; *P* = 0.038), OR = 1.56 (1.04–2.55), the frequency of the T allele being higher among cases (0.32) than controls (0.25). The risk allele (T) was identical in the two cohorts from Nigeria and Mali.

Finally, we analyzed the association of rs2227476 and rs2227473 with CM for the entire study population, by combining the case-control and familial data. Univariate analysis indicated that both SNPs were associated with CM (*P* ≤ 0.05) (Table 4). In multivariate binary regression analysis, the rs2227473 polymorphism remained significantly associated with CM (*P* = 0.004), with an estimated OR of 1.54 (1.15–2.07), whereas rs2227476 was not significantly associated with the disease in the combined sample set. These results confirm the association between *IL22* SNPs and the development of CM in both Nigerian and Malian children.

### The rs2227476 and rs2227473 *IL22* polymorphisms may both have functional effects

We carried out an *in silico* search for transcription factor binding sites encompassing rs2227476 and rs2227473 in the 5′ region of *IL22*. We found that GATA could potentially bind to DNA only in subjects carrying the T allele for rs2227476. We also found that rs2227473 was located within a putative binding site for the aryl-hydrocarbon receptor (AhR-ARNT) complex transcription factor. This complex is a potent inducer of *IL22* expression. These analyses suggest that rs2227476 and rs2227473, located in the promoter region of *IL22*, can modify *IL22* expression.

## Discussion

We identified susceptibility variants of the *IL22* gene associated with childhood CM, in two independent African populations. The association study was performed in two stages, with an initial case-control study population in Nigeria, and replication in a family-based population study in Mali. Two SNPs in the promoter region of *IL22* (rs2227476 and rs2227473) were associated with CM in both populations. The minor allele (the T allele) of these two SNPs increased the risk of CM in both populations. The rs2227473 SNP has been shown to influence susceptibility to pulmonary tuberculosis in the Chinese population^18^ and the onset of psoriasis before puberty^19^. The rs2227473 and rs2227476 SNPs, which are in LD in Nigeria (r^2^ = 0.72) and Mali (r^2^ = 0.5), are probably located within transcription factor binding sites and may therefore have an effect on IL-22 production. Functional assays on PBMCs showed that cells with a homozygous TT genotype or a heterozygous TC genotype for rs2227473 produced more IL-22 than cells with a homozygous CC genotype for this SNP^18^. Luciferase reporter assays showed that transcription rates were higher for the T allele than for the C allele^19^. *Ex vivo*-stimulated CD4 T cells from psoriasis patients carrying the T allele at rs2227473 produced more IL-22 than stimulated cells from patients carrying the C allele^19^. Consistent with these findings, we found that rs2227473 was located within a putative binding site for the transcription factor AhR-ARNT complex, as also reported by Nikomo *et al*.^19^. The identified risk allele, the T allele, should confer a 30% higher AhR-ARNT complex binding efficiency^19^. The aryl-hydrocarbon receptor interacts with diverse ligands and acts as a master regulator of IL-22 production^20^,^21^,^22^,^23^. It is essential for IL-22 production by Th17 cells^24^,^25^ and for Th17/Th22 polarization.

The rs2227473 variant may also alter putative binding sites for several transcription factors, including the *Plasmodium falciparum* PF14_0633, which has an AP2 domain^26^,^27^. This pathogen protein may target the host genes and regulate IL-22 expression^18^. These functional data and our genetic results suggest that the *IL22* SNPs, rs2227476 and rs2227473 may be causal for cerebral malaria. They also show that the T allele of rs2227473, which is associated with greater susceptibility to CM, is also associated with higher levels of IL-22 production. These results suggest that IL-22 is associated with an aggravation of malaria.

This aggregation may occur in several ways. IL-22 may promote the breakdown of the blood- brain barrier. Recent results strongly suggest that Th17 cells play a unique role in permeabilizing the human BBB to both soluble molecules and circulating CD4+ lymphocytes, through the action of IL-17 and IL-22^9^. IL-22 may exert its pathogenic effects in CM by acting on endothelial cells to promote the disruption of the BBB observed during this disease^3^,^28^. This hypothesis is supported by the finding that the IL-22R is expressed on BBB endothelial cells and determines the responsiveness of these cells to IL-22. There is also *in vivo* evidence to suggest that IL-22 may act as a pathogenic effector in CM by increasing the ability of Th17 cells to enter the central nervous system (CNS). These Th17 cells may then produce multiple mediators, including cytolytic enzymes, such as granzyme B, strongly favoring the development of encephalitis.

Alternatively, IL-22 may be involved in neuroinflammation. In addition to regulating the expression of antimicrobial peptides and defensins, IL-22 plays a role in the expression of various genes encoding molecules involved in inflammation. IL-22 is necessary and sufficient to promote tissue inflammation in several models of inflammatory disorders at barrier surfaces. It can stimulate the release of TNF-α and IL-8 from epithelial cells^29^ and may contribute to CM by enhancing inflammation in brain microvessels. The experimental autoimmune encephalomyelitis (EAE) model of multiple sclerosis (MS) is often used in studies of neuroinflammatory disease mechanisms. This model seems to have several features in common with CM. *In vivo* imaging in animals with experimental cerebral malaria has shown that the vascular damage observed, including BBB disruption, can be attributed to inflammatory processes^30^, as already reported for multiple sclerosis lesions. IL-22 levels increase during the induction of EAE and the peak of the disease, whereas a decrease in these levels is associated with recovery in the rat EAE model^31^.

The lack of association between CM and *IL22RA2* polymorphisms suggests that IL-22BP may not regulate IL-22 production in CM. IL-22BP may not be produced at the site of IL-22 action. By contrast, IL-22 and other inflammatory cytokines, such as IL-17, are produced in sufficient amounts to increase disease severity^9^,^32^,^33^.

IL-22 has also been shown to have a deleterious effect in the pathogenesis of West Nile encephalitis, in which it may promote neuroinvasion by the virus^16^. High IL-17 and IL-22 levels have also been observed in the cerebrospinal fluid and plasma of patients developing Guillain-Barré Syndrome^17^, and are thought to cause disruption of the BBB and local inflammation of the peripheral nervous system.

Experimental blood-stage malarial infections have shown that IL-22 is probably protective in the liver. Indeed, protection from fatal liver tissue damage has been observed during primary *Plasmodium chabaudi* infection^34^, consistent with the known hepatoprotective function of IL-22^12^,^35^,^36^,^37^.

In conclusion, we have shown that variants of the *IL22* gene may play an important role in the pathogenesis of CM and that these variants of *IL22* are associated with an aggravation of malaria. The rs2227473 risk allele for CM, the T allele, is associated with higher levels of IL-22 production, suggesting that IL-22 contributes to CM. We suggest that IL-22, acting in synergy with IL-17A, may have a deleterious effect in cerebral malaria, through the direct or indirect promotion of BBB permeability and brain inflammation, whilst protecting infected individuals against blood-stage infections and fatal liver tissue damage. It has been suggested that IL-22 acts as a gatekeeper, maintaining immune homeostasis at barrier surfaces through the induction of innate antimicrobial immunity^6^,^13^,^38^.

## Materials and Methods

### Ethics statement

The internationally recognized joint ethics committee of the College of Medicine of the University of Ibadan and the University College Hospital in Ibadan approved the Nigerian case-control study, which was part of a larger case-control study on severe malaria in children. The parents or guardians of children from Ibadan, Nigeria gave written informed consent for the participation of their children in the study. Consent forms translated into Yoruba were provided where appropriate. The Malian study was performed with the approval of the local ethics committees of the Faculty of Medicine, Pharmacy and Odonto-Stomatology of the University of Bamako, and written informed consent was obtained from all parents. All experimental methods were carried out in accordance with the approved guidelines.

### Study participants

We performed an association study for CM in two independent study populations from Africa: one cohort of children from the city of Ibadan, Nigeria and one cohort from Bamako, Mali.

All participating children from the city of Ibadan were recruited under the auspices of the Childhood Malaria Research Group (CMRG) of the Department of Pediatrics at University College Hospital (UCH), Ibadan, Nigeria. These children were part of a larger prospective case-control study of severe malaria described elsewhere^39^,^40^,^41^. Briefly, we recruited children between the ages of six months and 13 years with severe malaria defined according to WHO criteria^42^,^43^. Cerebral malaria (CM) was defined as a state of unarousable coma (Blantyre coma score ≤2) lasting for at least one hour, accompanied by parasitemia with asexual stages of *Plasmodium falciparum* and normal findings for cerebrospinal fluid. Children with CM were also considered to have severe malarial anemia (SMA) if they had a packed cell volume (PCV) of less than 16%. None of the recruited patients died. The community control (CC) group consisted of age-matched parasite-negative children from the same community. These children had no patent symptoms of illness and were clinically healthy. The case-control study was performed on a population of 115 children with CM and 160 CCs. The mean age of the children with CM was 4.5 years (range: 10 months - 13 years), and 47.8% of these patients were female. The CC children had a mean age of 6 years (range: 6 months - 13 years), and 48.1% of these controls were female.

Malian children with CM were recruited through the Pediatrics Department of Gabriel Toure Hospital in Bamako, Mali. These hospitalized children were selected during a larger prospective study recruiting both children with CM and SMA and their parents^44^,^45^,^46^,^47^. The criteria used to define CM were identical to those for the Nigerian study: coma with a Blantyre score ≤ 2 and a thick blood smear positive for *P. falciparum*. Meningitis was ruled out by lumbar puncture. A full description of the recruitment of Malian children is provided elsewhere^45^. In total, 240 nuclear families were recruited. The mean age of the children with CM was 6 years (range: 10 months to 15 years), and 49.6% of these patients were female.

The basic characteristics of the two study populations have been described elsewhere^48^. Prospective subject selection allowed for careful phenotypic characterization and matching between the two populations.

Genomic DNA was extracted from the peripheral blood leukocytes of subjects from the Nigerian and Malian cohorts, as previously described^45^.

### SNP selection and genotyping

Tagged SNPs were selected for the *IL22* (MIM 605330) and *IL22RA2* (MIM 606648) genes, including 5 kb on either side of these genes. SNPs with a minor allele frequency ≥0.05 were selected from the 1000 Genomes YRI database, and PLINK ( http://pngu.mgh.harvard.edu/~purcell/plink/index.shtml) was used to determine r^2^ values for correlations between SNPs. We selected one SNP per correlation bin, using an r^2^ cutoff of 0.8, and the singleton SNPs. This analysis resulted in the selection of 46 SNPs, 19 for *IL22* and 27 for *IL22RA2*. We genotyped these polymorphisms in the Nigerian population, which consisted of 115 children with CM and 160 CCs, none of whom were related. Genotyping was performed with a custom-designed Sequenom IPLEX Assay, in accordance with the manufacturer’s instructions. SNPs were excluded if they had a call rate <0.90 (Table 1).

The SNPs selected from these analyses (rs1012356 and rs2227476) were then genotyped in 240 nuclear families from Mali. We also genotyped all the SNPs located within a 1 Mb window and in LD with rs2227476 (r^2^ > 0.6 assessed by PLINK) according to the 1000 Genomes YRI database, in the Malian sample. In total, we genotyped 14 SNPs of *IL22* or the surrounding regions (rs6581806 r^2^ = 0.82, rs11177141 r^2^ = 0.82, rs7954576 r^2^ = 0.82, rs4913430 r^2^ = 0.82, rs6581805 r^2^ = 0.82, rs4913429 r^2^ = 0.82, rs2127537 r^2^ = 0.82, rs7301738 r^2^ = 0.82, rs7298302 r^2^ = 0.82, rs12307915 r^2^ = 0.82, rs2227481 r^2^ = 0.80, rs7298415 r^2^ = 0.72, rs2227478 r^2^ = 0.72 and rs2227473 r^2^ = 0.69) in the Malian cohort, with either the IPLEX Assay (Sequenom) or TaqMan SNP Genotyping Assays (Applied Biosystems).

### Statistical analysis

A Chi^2^ test was used to determine whether the genotype distribution in parents and controls conformed to Hardy-Weinberg equilibrium. None of the polymorphisms deviated from Hardy-Weinberg equilibrium in tests with a threshold for significance of *P* = 0.05 carried out in GenePop software.

Univariate and multivariate analyses of the SNPs were carried out with SPSS (statistical software version 20), to assess the association between the SNPs and CM in unrelated Nigerian subjects. Differences were considered to be significant if the *P*-value obtained in a two-tailed test was <0.05. We first tested for associations between individual SNPs and the CM phenotype (univariate analysis) and we then analyzed the association between combinations of SNPs (associated or with a trend towards association with CM) and CM by logistic regression analysis (multivariate analysis).

We used the family-based association test package (FBAT; version 1.7)^49^ for association tests in nuclear families from Mali. A SNP was considered to be replicated if the association analysis yielded a *P*-value < 0.05 in two-tailed tests, with the same risk allele as identified in the Nigerian population.

A case-pseudo control data set was used and a conditional logistic regression analysis was performed, as previously described^47^,^50^,^51^, to estimate the odds ratio (OR) in Malian subjects. We also performed a combined conditional logistic regression analysis on both the discovery case-control cohort and the replication family-based cohort.

### *In silico* analysis

We used the TFsearch program ( http://www.cbrs.jp/research.db/TFSEARCH.html) to predict the potential transcription factor binding sites for *IL22*. We also used the RAVEN (regulatory analysis of variation in enhancers) system ( http://www.cisreg.ca) to detect and characterize regulatory sequence variation^52^. This approach identifies genetic variants likely to influence gene regulation and combines phylogenetic footprinting with the reported effects of genetic variation on putative transcription factor binding sites.

## Additional Information

**How to cite this article:** Marquet, S. *et al*. A Functional IL22 Polymorphism (rs2227473) Is Associated with Predisposition to Childhood Cerebral Malaria. *Sci. Rep.* **7**, 41636; doi: 10.1038/srep41636 (2017).

**Publisher's note:** Springer Nature remains neutral with regard to jurisdictional claims in published maps and institutional affiliations.

## Figures and Tables

**Table 1:** List of the 46 TagSNPs included in the discovery study.

Gene (chr)	TagSNP	Position	Minor Allele	Major Allele	MAF	Call rate %	HWp
IL22	rs1179246	68640583	A	C	0.39	98.2	0.58
(12)	rs2227507	68642646	C	T	0.06	97.4	0.54
	rs1179249	68644780	T	G	0.14	98.7	0.1
	rs2227495	68644726	G	A	0.14	96.9	0.77
	rs2227485	68647713	A	G	0.49	96.1	0.08
	rs2227476	68648816	T	A	0.32	95	0.1
	rs11177135	68649606	A	G	0.11	91	0.08
	rs17105141	68650108	G	A	0.18	98.2	0.46
	rs58254691	68640611	T	G	0.08	92	1
	rs1182844	68641532	A	T	0.31	94	0.09
	rs2227505	68643267	T	C	0.06	99.7	0.1
	rs1012356	68644618	T	A	0.47	98.7	0.2
	rs1179251	68645051	G	C	0.45	98.5	0.64
	rs2046068	68645975	G	T	0.12	97.7	0.49
	rs2227491	68646521	T	C	0.41	99	0.46
	rs7139027	68649389	T	C	0.14	97.1	0.42
	rs115479956*	68649678	G	A	0.05	/	/
	rs7302661	68651459	T	C	0.08	98.5	0.29
	rs4913285	68652532	T	C	0.15	97	0.2
IL22RA2	rs28362176	137465084	A	G	0.05	93.2	0.26
(6)	rs79938603	137461160	A	G	0.08	95.3	0.39
	rs28385776	137473682	A	G	0.05	99.7	0.47
	rs85462	137463154	G	C	0.08	97.9	0.09
	rs79745323*	137463396	A	G	0.10	/	/
	rs17066102*	137464837	C	G	0.25	/	/
	rs10484798	137470756	C	T	0.30	97.9	0.82
	rs7774349	137475858	T	C	0.05	100	
	rs202568	137471184	T	C	0.11	98.2	0.29
	rs28385776	137473682	A	G	0.05	99.7	0.47
	rs28385767	137481894	A	G	0.24	99.5	0.2
	rs1040622	137483258	C	T	0.07	98.7	0.72
	rs73560046	137493433	T	C	0.1	99	1
	rs9494683	137492101	T	C	0.16	95.6	0.29
	rs742931	137495707	G	T	0.23	96.4	0.72
	rs202563	137461492	A	G	0.41	98.5	0.81
	rs156751	137463294	T	C	0.05	99.5	0.48
	rs11154911	137466364	T	C	0.05	99.5	0.48
	rs114513054	137467551	G	C	0.08	99.2	0.86
	rs28362171*	137469118	C	G	0.07	/	/
	rs7750867	137470186	T	C	0.07	97.1	0.43
	rs28362169	137471111	T	C	0.08	99.5	0.92
	rs74413263	137482668	G	A	0.06	91.9	0.44
	rs73560035*	137483523	T	C	0.05	/	/
	rs113837410*	137487743	C	T	0.08	/	/
	rs28362847	137492804	A	G	0.33	99.5	0.89
	rs6570137	137498645	T	C	0.1	97.1	0.64

^*^These SNPs were excluded due to a low call rate.

^a^Position according to human hg19 genome coordinates.

^b^MAF (minor allele frequency) was estimated for the YRI population according to 1000 Genomes project data.

^c^The call rate indicates the efficiency of genotyping.

^d^HWp (Hardy-Weinberg *p* value) estimated with Genepop.

**Table 2:** Association of *IL22* SNPs with CM in the Nigerian population-based study.

	Bin	SNP	Minor Allele	MAF^a^	Genotypes	Controls %	Cases %	OR	95% CI	*P*^b^
Univariate analysis	I	rs1179251	G	0.42	GG, CC	47.8	59.6	1.61	1.02–2.64	0.057
	II	rs1012356	T	0.47	TT	19	31.2	1.93	1.10–3.41	0.022
	III	rs2227476	T	0.24	TT, TA	38.2	53.3	1.85	1.11–3.08	0.018
	IV	rs4913285	T	0.10	TC, TT	18.9	28.8	1.74	1.02.3.09	0.056
Multivariate analysis	II	rs1012356	T	0.47	TT			2.12	1.1–3.9	0.016
	III	rs2227476	T	0.24	TT, TA			2.08	1.2–3.6	0.007

Severe cases were defined as children with cerebral malaria (CM, *n* = 115).

The controls were community controls (CC, *n* = 160).

^a^MAF (minor allele frequency) was estimated from Nigerian community control data.

^b^All the *P*-values reported are for two-tailed tests.

OR, odds ratio; CI, confidence interval.

**Table 3:** Association of *IL22* SNPs with CM in the Malian family-based study.

Bin	SNP	Risk Allele	Freq^a^	Model	Informative families	Transmitted alleles		OR (95% CI)	*P- Value*^b^	*Pp*_*-*_*Value*^c^
						Observed	Expected			
II	rs1012356 (A/T)	T	0.49	ADD	168	177	163.3	1.46 (1.03–2.07)	0.06	0.06
III	rs2227476 (A/T)	T	0.23	REC	37	19	11.75	1.56 (1.09–2.17)	0.008	0.010

240 trios (two parents and one child with CM) were included in the family-based association study.

^a^Frequency of the risk allele in the Malian cohort.

^b^Two-tailed test relative to the risk allele (FBAT).

^c^FBAT *P*-value obtained in the FBAT permutation test (100,000 permutations).

OR, odds ratio; CI, confidence interval; ADD, additive; REC, recessive.

**Table 4:** Association of *IL22* SNPs with CM in the Nigerian and Malian cohorts.

	Bin	SNP	Risk Allele	Freq^a^	Model	Genotypes	OR (95% CI)	*P Value*^b^
Univariate analysis	III	rs2227476 (A/T))	T	0.24	ADD	TT, TA	1.29 (1.04–1.73)	0.05
	III	rs2227473 (T/C)	T	0.25	ADD	TT, TC	1.46 (1.10–1.94)	0.008
Multivariate analysis	III	rs2227473 (T/C)	T	0.25	ADD	TT, TC	1.55 (1.15–2.07)	0.004

^a^Frequency of the risk allele in our study population.

^b^Two-tailed test relative to the risk allele.

OR, odds ratio; CI, confidence interval; ADD, additive.
